# Serological evidence of continued Japanese encephalitis virus transmission in Singapore nearly three decades after end of pig farming

**DOI:** 10.1186/s13071-019-3501-0

**Published:** 2019-05-17

**Authors:** Grace Yap, Xiao Fang Lim, Sharon Chan, Choon Beng How, Mahathir Humaidi, Gladys Yeo, Diyar Mailepessov, Marcella Kong, Yee Ling Lai, Chiharu Okumura, Lee Ching Ng

**Affiliations:** 1 Environmental Health Institute, National Environment Agency, Singapore, Singapore; 2 Department of Biological Sciences, National University of Singapore, Singapore, Singapore; 3 National Parks Board, Singapore, Singapore; 4 Wildlife Reserves Singapore, Singapore, Singapore

**Keywords:** Japanese encephalitis virus (JEV), Singapore, Wild pigs, Migratory birds, Wild birds

## Abstract

**Background:**

Singapore used to report an annual average of 14 cases of Japanese encephalitis, but ever since the abolishment of pig farms in the early 1990s, the local incidence rate for Japanese encephalitis virus (JEV) infections has reduced drastically. Studies done in the early 2000s demonstrated the presence of JEV-specific antibodies in animals such as wild boars, dogs, chickens and goats on the offshore island and peripheral parts of the Singapore, indicative of prior JEV exposure. A JEV wildlife and sentinel chicken surveillance system was initiated in 2010 through to 2017 to study the animal host seroprofiles.

**Results:**

A total of 12/371 (3.23%) of resident bird samples, 24/254 (9.45%) of migratory bird samples and 10/66 (15.16%) of wild boar samples were positive for the presence of JEV antibodies. Seroconversions in sentinel chickens were observed at two time points. Through this study, two sites with active transmission of JEV amongst avian or porcine hosts were identified.

**Conclusions:**

JEV transmission in animal hosts has continued despite the phasing out of pig farming nearly thirty years ago; however, the public health risk of transmission remains low. Environmental management for mosquito population remains key to keeping this risk low.

## Background

Japanese encephalitis is endemic in many Asian countries and contributes to a large disease burden, with approximately 30,000 to 50,000 reported cases, and 10,000 deaths annually [1]. Epidemics of encephalitis have been described since the 1870s in Japan, with the first isolation of the Japanese encephalitis prototype Nakayama strain in 1935 from the brain of a fatal human case. Japanese encephalitis virus (JEV) has subsequently been isolated in China and the Asian subcontinent, all of South East Asia and the Pacific Rim, and reached the northern regions of Australia in 1998 [2, 3]. JEV is divided into five genotypes (GI to GV) with GIII as the source of numerous epidemics throughout history. However, there have been reports of replacement of GIII with GI in the recent years [4, 5]. This genotype replacement was hypothesized to be linked to viral genetic determinants related to selective advantages in the mosquito vectors.

Singapore used to report an annual average of 14 cases of JEV until the abolishment of pig farms in 1992 [6–11]. JEV transmission involves pigs as the amplifying host and ardeid birds as the reservoir host, with *Culex tritaeniorhynchus* as the primary vector, and humans as the accidental dead-end host [12]. After 1992, the local incidence of JEV infections reduced drastically. From 1991 to 2000 there were three imported cases of JEV. In 2001, two local cases were reported and the latest case, in May 2005, reported a 53-year-old man, whose serum sample tested positive for JEV [13]. JEV-specific antibodies were also detected in animals such as wild boars, dogs, chickens and goats in 2002 and 2004 on the offshore islands and rural parts of Singapore [14, 15]. These antibody profiles served as an important indicator that JEV transmission could be active in those areas among the avian and porcine hosts. In countries where JEV transmission is apparent, animals similarly display the presence of neutralizing antibodies in their blood [16, 17]. The Eurasian wild pigs (*Sus scrofa* Linnaeus), which were once thought to be extinct in Singapore have re-colonized areas such as the Central Catchment Nature Reserve (CCNR) and the western catchment areas in the past decade. It is estimated that over 500 wild pigs inhabit the CCNR. Small population clusters are also found on the eastern side of Singapore [18]. The expansion of the appropriate amplifying host population may contribute to the continued risk of JEV transmission in Singapore.

A plausible source of JEV is migratory birds which can serve as viral reservoirs. Over 90 bird species have been identified as amplifying and reservoir hosts of JEV. Among them, egrets (*Egretta garzetta*) and herons (*Nycticorax nycticorax*) from the family Ardeidae are particularly susceptible to JEV infection, and are known to develop high viral titers [19, 20]. During each migratory season from September to March, birds fly along the East-Asia Australasian Flyway (EAAF) and stopover at a Singapore’s Nature Reserve (NR). These birds fly from as far north as the Arctic Circle, across East and South East Asia, and journey southwards to Australia and New Zealand. Migratory birds are thought to be one of the factors involved in the introduction of JEV GI from South East Asia in the early 1990s to more temperate regions such as northern Vietnam and eastern Asia (Japan and Korea) [3, 21].

This study aims to give an updated seropositivity profile of JEV among animals in Singapore, particularly in local and migratory birds, and in wild boar samples collected from 2011 to 2017.

## Methods

### Collection of migratory/resident bird sera and dead bird carcases

Nature Reserve (NR) houses a variety of resident birds, and is also visited by both long- and short-distance migratory birds. NR has an existing bird ringing programme to collect bio-data from the migratory birds that visit this pit-stop. Tagging onto this programme that has been on-going since 1990, 254 blood samples were taken from the migratory birds that were caught during the migratory session (every September to March) from 2010 to 2017. Jugular vein blood samples of not more than 1% body weight of birds were drawn aseptically into EDTA tubes by avian veterinarians. After sampling, an equivalent volume of 10% glucose was injected for replenishment. Blood samples were transferred on ice to the Environmental Health Institute, a public health laboratory under the National Environment Agency of Singapore. The plasma was separated by centrifugation at 4000 rpm for 4 min and kept at − 80 °C until further processing.

Through a donor bird programme at the Avian Sanctuary (AS) where the public can submit injured birds found elsewhere in the country, 372 bird serum samples were obtained during the same period. The collection also included 1024 dead bird carcasses. These carcasses were dissected, and their organs (heart, liver, spleen, lungs, kidneys and brain) were stored at − 80 °C for bio-banking purposes.

### Collection of wild boar sera

Sixty-six wild boar blood samples were collected as part of wild boar population management programme in Central Catchment Nature Reserve (CCNR) in 2014, where wild boar activity was previously documented. Animals’ physical characteristics such as size and weight were recorded. Blood was kept and transferred to the laboratory on ice. The plasma was separated by centrifugation at 4000 rpm for 4 min and kept at − 80 °C until further processing.

### RNA extraction and real-time RT-PCR

A total of 1024 bird brain samples were each homogenized in 500 µl of virus transport media (Copan Diagnostics, California, USA) using a Mixer Mill MM 400 (Retsch Technology GmbH, Haan, Germany). A volume of 140 µl of viral RNA was extracted from each brain specimen and blood sample by using a QIAamp viral RNA mini kit (Qiagen, Hilden, Germany) according to the manufacturer’s instructions.

A duplex JEV and West Nile (WN) real-time reverse transcription polymerase chain reaction (RT-PCR) protocol was optimised based on protocols by Scherret et al. [22] and Santhosh et al. [22, 23]. A pan-flavivirus RT-PCR adopted from Yang et al. [26] was also performed. Extracted RNA was subjected to both PCR reactions. Primers from Yang et al. [26], Scherret et al. [22] and Santhosh et al. [23] were utilized. Briefly, the reaction occurred in a final volume of 20 µl, comprising 5 µl of RNA template and a concentration of 10 µM for each primer in a Lightcycler® 2.0 (Roche Diagnostics GmbH, Mannheim, Germany). The cycling conditions were as follows: reverse transcription at 50 °C for 20 min, inactivation at 95 °C for 15 min and subsequently 40 cycles of 94 °C for 15 s, 55 °C (JE-WN) or 58 °C (Flaviviruses) for 30 s and 72 °C for 30 s. Melting curve analysis was conducted at 65 °C for 15 s continued with a cooling step at 37 °C for 20 s for amplified products accuracy verification.

### Sentinel chicken for passive surveillance in NR

From 2013 to 2018, a sentinel chicken surveillance was established at NR. As part of this surveillance, three to five sentinel chickens (*Gallus domesticus*) were deployed at NR for monitoring of specific zoonotic diseases, particularly arboviruses (arthropod-borne viruses). As mosquito-borne viruses such as JEV utilize birds in their transmission cycles, chickens are good sentinels and can act as an early warning system [24, 25]. Monthly blood samples were collected and processed. All procedures were approved and carried out in accordance to the Environmental Health Institute’s Institutional Animal Care and Committee (IACUC) guidelines (IACUC005). The plasma samples were subsequently subjected to immunofluorescence assay (IFA) to determine for the presence of seroconversion for JEV.

### Detection of JEV antibodies in resident/donor birds, wild boars and sentinel chickens using immunofluorescence assay (IFA)

The plasma samples separated from mammal and bird blood were subjected to IFA assay to determine the seropositivity. JEV (Nakayama strain, courtesy of Dr Y. C. Chan) was cultured in Vero cells (ATCC CCL-81) in the biosafety level 3 (BSL3) laboratory at the Environmental Health Institute. Immunofluorescence slides were made, inactivated and transferred out of the BSL3 to BSL2 where the IFA was performed. Animal plasma was diluted 1:50 in 5% skim milk (Oxoid, Kansas, USA) and applied onto the JEV IFA slides. Incubation was performed in a humidifier at 37 °C for 30 min before washing with 1× PBS. Secondary antibody anti-bird Ig-FITC (Bethyl Laboratories, Texas, USA) or anti-pig Ig-FITC (Bethyl Laboratories) was applied onto the slides, depending on the animal sera tested. Incubation and washing were repeated before visualization of fluorescence was performed.

### Plaque reduction neutralization assay

Samples that were positive in the IFA were subjected to plaque reduction neutralization assay (PRNT). PRNT, adapted from Nemeth et al. [26–28], was carried out in 24-well plates seeded with baby hamster kidney cells (BHK21, ATCC CCL-10) at 90% confluence. The animal plasma samples were heat inactivated at 56 °C for 30 min before use. Samples were serially diluted from 1:5 to 1:500, and 100 µl of each dilution was incubated with 100 µl of JEV virus (20 pfu/well) (making the final dilution of the sera 1:10, 1:100 and 1:1000, respectively) at 37 °C for 1 h. The antibody and virus mixtures were then added to the wells of the 24-well plates containing the BHK cells. The medium was aspirated after 1 h of incubation at 37 °C. BHK cells were overlaid with 1 ml of 1% carboxymethylcellulose in RPMI medium supplemented with 2% FCS. The plates were incubated at 37 °C for approximately 5–6 days with 5% CO_2_. Subsequently, each well was fixed with formaldehyde (20%) for 30 min and stained with napthol blue black stain solution. PRNT_80_ was used to differentiate any cross-neutralizating antibodies from the same JEV serocomplex. The PRNT_80_ titre was calculated by counting plaques and reporting the titre as the reciprocal of the last serum dilution to show 80% reduction of control plaques (≤ 4 plaques) based on approximately 20 pfu of virus added to each sample.

## Results

A total of 371 donor bird sera were screened for JEV antibodies by IFA, followed by the confirmation of neutralizing antibodies by PRNT. The panel of birds included species such as buzzards, bitterns, eagles, egrets, flamingos, hawks, herons, hornbills, kingfishers, koels, owls, pigeons, pittas and turacos. Twelve out of 371 (3.23%) registered JEV neutralizing antibodies, with an end point titre ranging from 10^1^–10^3^ (Table 1).

**Table 1:** JEV seropositivity among donor birds, wild boars and sentinel chickens. Seropositivity was confirmed by PRNT after IFA screening. PRNT_80_ end point titre was determined by counting plaques and reporting the titre as the reciprocal of the last serum dilution to show 80% reduction of control plaques (≤ 20 plaques) counted based on the 100 pfu of virus added to each sera sample

Sample ID	Bird species	Sex/Age group	Location	Date collected	PRNT_80_ (end-point titer)
DB2	Crested goshawk (*Accipiter trivirgatus*)	na	Bukit Panjang	March 2011	10^3^
DB3	White-bellied sea eagle (*Haliaeetus leucogaster*)	na	Changi Airport	March 2011	10^2^
DB4	Changeable hawk-eagle (*Nisaetus cirrhatus*)	na	Jurong West HDB	April 2011	10^2^
DB9	Crested serpent eagle (*Spilornis cheela*)	na	Not available	May 2011	10^3^
DB13	Oriental honey-buzzard (*Pernis ptilorhyncus*)	na	Japanese Garden	May 2011	10^2^
DB14	Black-crowned night heron (*Nycticorax nycticorax*)	na	Pasir Ris Park	May 2011	10^1^
DB20	White-bellied sea eagle (*Haliaeetus leucogaster*)	na	Lim Chu Kang MRT	Not available	10^2^
DB31	White-bellied sea eagle (*Haliaeetus leucogaster*)	na	Upper Seletar Reserve	April 2011	10^1^
DB38	White-bellied sea eagle (*Haliaeetus leucogaster*)	na	Tuas	March 2012	10^1^
DB41	Cattle egret (*Bubulcus ibis*)	na	Jurong East	July 2012	10^3^
DB59	Little egret (*Egretta garzetta*)	na	Japanese Garden	October 2012	10^1^
DB71	Heron (not identified to species)	na	Avian Sanctuary	Not available	10^2^
MP10	Wild boar (*Sus scrofa*)	na	Central Catchment Nature Reserve	Mar 2013	10^2^
MP12	Wild boar (*Sus scrofa*)	na	Central Catchment Nature Reserve	April 2013	10^1^
MP19	Wild boar (*Sus scrofa*)	na	Central Catchment Nature Reserve	June 2013	10^2^
MP27	Wild boar (*Sus scrofa*)	na	Central Catchment Nature Reserve	July 2013	10^1^
MP32	Wild boar (*Sus scrofa*)	Male; juvenile	Central Catchment Nature Reserve	September 2013	10^1^
MP33	Wild boar (*Sus scrofa*)	Male; adult	Central Catchment Nature Reserve	December 2013	10^1^
MP38	Wild boar (*Sus scrofa*)	Male; juvenile	Central Catchment Nature Reserve	December 2013	10^2^
MP41	Wild boar (*Sus scrofa*)	Male; juvenile	Central Catchment Nature Reserve	December 2013	10^2^
MP44	Wild boar (*Sus scrofa*)	Female; adult	Central Catchment Nature Reserve	January 2014	10^2^
MP45	Wild boar (*Sus scrofa*)	Female; piglet	Central Catchment Nature Reserve	January 2014	10^3^
SC281013Gr	Chicken (*Gallus domesticus*)	Female; adult	Nature Reserve	October 2013	10^1^
SC281013Pu	Chicken (*Gallus domesticus*)	Female; adult	Nature Reserve	October 2013	10^1^
SC281013Ye	Chicken (*Gallus domesticus*)	Female; adult	Nature Reserve	October 2013	10^2^
SC031114Bl	Chicken (*Gallus domesticus*)	Female; adult	Nature Reserve	November 2014	10^1^
SC031114Re	Chicken (*Gallus domesticus*)	Female; adult	Nature Reserve	November 2014	10^1^
SC031114Pu	Chicken (*Gallus domesticus*)	Female; adult	Nature Reserve	November 2014	10^2^

*Abbreviation*: na, not available

Besides ardeid birds (black-crowned night heron, cattle egret and little egret), which are the typical avian reservoirs of JEV, raptors (crested goshawk, honey buzzard and white-bellied sea eagle) also showed JEV neutralizing antibodies. The common birds in this collection (crows, mynahs, pigeons, doves, herons, egrets and bitterns) are considered as part of the native bird community.

A total of 254 plasma samples were collected from migratory birds which included plovers (golden Pacific plovers, *Charadrius mongolus*; Mongolian plovers, *Pluvialis fulva*) and common redshanks (*Tringa tetanus*). Out of this, 24 (9.45%) samples collected from 2010 to 2012 registered neutralizing antibodies towards JEV (Table 2).

**Table 2:** JEV seropositivity rates of migratory birds from 2010 to 2017. A total of 254 samples were screened and 24 (9.45%) registered neutralizing antibodies towards Japanese encephalitis virus, with PRNT _80_ end point titer of 10^1^ to 10^3^

Bird species	Seropositivity rate (%)^a^							
	2010	2011	2012	2013	2014	2015	2016	2017
Plovers	11.1 (1/9)	9.1 (3/33)	18.2 (2/11)	0 (0/2)	0 (0/4)	0 (0/0)	0 (0/6)	0 (0/4)
Common redshank	50.0 (5/10)	69.2 (9/13)	15.4 (4/26)	0 (0/9)	0 (0/38)	0 (0/61)	0 (0/10)	0 (0/18)

^a^Seropositive sera/total sera tested

Of 66 wild boar samples screened, 10 (15.16%) plasma samples had JEV neutralizing antibodies, with an end point titre ranging from 10^1^–10^3^ (Table 1).

Three to five sentinel chickens were placed in NR since 2013 for passive surveillance of specific zoonotic diseases. Seroconversion was observed in October 2013 and November 2014, with an end point titre ranging from 10^1^ to 10^2^ (Table 1). There was no seroconversion from then until the sentinel chicken surveillance was ceased in August 2018.

None of the sera or tissues was positive for JEV RNA by real-time RT-PCR.

## Discussion

With the abolishment of pig farms in 1992, the transmission of JEV in Singapore has seemingly ceased. However, various studies on the animal seroprofiles both on mainland Singapore and offshore islands from 2001 have revealed that JEV may still be actively transmitting among native animals. In this study, we provide an updated seropositivity profile of JEV among animals in Singapore. Besides considering the wild birds as effective sentinels, sentinel chickens were also deployed at NR to determine if there was any transmission amongst wild birds.

Although no JEV RNA was detected in any of the avian samples, JEV neutralizing antibodies were detected in ardeid birds and raptors. Egrets and herons are known avian reservoirs for JEV, but not raptors. Raptors, however, are susceptible to West Nile Virus infection (WNV), a flavivirus in the JEV antigenic complex [29]. It is interesting to note that raptors, such as the honey buzzard and white-bellied sea eagle, are non-terrestrial birds and roost in tall trees. Therefore, they are usually not available for feeding by *Culex tritaeniorhynchus*, the main vector of JEV, which is a terrestrial biter. This suggests the presence of a canopy biter, which is involved in the horizontal transmission of JEV to raptors [28]. Alternatively, transmission *via* consumption of infected prey could be a possible route, in the same manner as the transmission of WNV among birds of prey such as owls, falcons, ospreys, vultures, and bald and golden eagles.

Out of the 24 migratory birds that had neutralizing antibodies towards JEV, six were Mongolian plovers (*Charadrius mongolus*) and the rest were common red shanks (*Tringa totanus*). Interestingly, JEV seropositive birds were only detected between 2010 and 2012, during which notable JEV outbreaks in this region occurred in East Asia, namely China and Korea [30, 31]. From 2013 to 2017, none of the birds sampled were seropositive. In the same period, JEV outbreaks were largely reported in Southeast Asian countries such as Philippines and Myanmar [32, 33]. The pattern of seropositivity detected here could be related to the movement of the birds. These migratory birds of the EAAF are known to breed in the northern regions, e.g. Mongolia, Russia, Tibet, and Alaska, and over-winter in East and Southeast Asia, Australia and New Zealand [34, 35]. However, the specific stopover points are not well known. Further studies to map out the flight path of these birds are needed.

The seroconversion of sentinel chickens placed in NR in October 2013 and November 2014 suggested active JEV transmission within the reserve during this time period. In this setting where ardeid birds and suitable mosquito vectors coexist, and migratory birds visit annually, the ecology is supportive of JEV transmission. However, the source of JEV infection remains undetermined. Over 28,000 *Culex* mosquitoes were screened, and no infected mosquitoes were found at NR throughout the duration of our study (Table 3). There were no human cases of JEV infection in Singapore reported during the same period. This is perhaps because the mosquito vectors are nocturnal and NR is closed at night; there is therefore little opportunity for an infected mosquito to encounter a human host. Moreover, NR is a heritage park with a rich biodiversity, including a sizable avian population which provide blood meals to these mosquitoes. No JEV cases have been detected through EHI’s febrile disease surveillance programme which has screened over 10,000 dengue negative samples since 2014 (unpublished data). The absence of human cases may also be due to high asymptomatic rate, and low suspicion index for JEV infections by healthcare practitioners in a country where JEV is not known to be endemic. Nevertheless, environmental management to keep mosquito population low remains the key in minimizing transmission of mosquito-borne diseases.

**Table 3:** *Culex* species and total number of mosquitoes trapped at Nature Reserve

Mosquito species	No. trapped
*Culex* (*Culex*) *bitaeniorhynchus*	166
*Culex* (*Culex*) *gelidus*	25
*Culex* (*Culex*) *quinquefasciatus*	307
*Culex* (*Culex*) *sitiens*	1559
*Culex* (*Culex*) *tritaeniorhynchus*	176
*Culex* spp. (*vishnui* subgroup)	26,146
Total *Culex* spp.	28,379

Neutralizing antibodies were detected in 10 out of 66 wild boars. We observed that JEV-specific antibodies were also detected in juveniles and hence, postulated that these were maternal antibodies. Presence of antibodies in adults suggests that transmission of JEV is active in this location. No JEV RNA was detected in the wild boars, indicating absence of viremia at the point of blood collection. This phenomenon was also observed in countries that are known to be endemic for JEV, such as Japan [16, 17]. Direct pig-to-pig transmission of JEV may also facilitate the virus circulation within the wild boar population [36].

## Conclusions

Our results suggest active transmission of JEV in two areas (Central Catchment Nature Reserve and NR) in Singapore involving avian and porcine hosts, nearly thirty years after the abolishment of pig farming. Further risk assessment studies will focus on identifying spatial risk of JEV in Singapore through surveillance of vector and animal hosts.

## Data Availability

Data supporting the conclusions of this article are provided within the article. The datasets used and/or analysed during the present study are available from the corresponding author upon reasonable request.

## Abbreviations

NR: nature reserve
AS: avian sanctuary
CCNR: Central Catchment Nature Reserve
EAAF: East-Asia Australasian flyway
